# Polyvinylpyrrolidone can effectively improve the efficiency of resiquimod in sorting bovine Y sperm

**DOI:** 10.5713/ab.24.0738

**Published:** 2025-03-31

**Authors:** Fei Huang, Peng Niu, Hui-min Qu, Hong Cheng, Jie-ru Wang, Jia-jia Suo, Jie Wang, Di Fang, Qing-hua Gao

**Affiliations:** 1 College of Life Science and Technology, Tarim University, Xinjiang, China; 2 College of Animal Science and Technology, Tarim University, Xinjiang, China; 3 Key Laboratory of Tarim Animal Husbandry Science and Technology, Xinjiang Production & Construction Corps, Xinjiang, China

**Keywords:** Bovine X /Y Sperm, Gender Control, Polyvinylpyrrolidone, Resiquimod

## Abstract

**Objective:**

The X/Y sperm separation technique plays a crucial role in gender control. The objective of this experiment is to investigate the effect of polyvinylpyrrolidone (PVP) concentration (A: 0%, B: 1%, C: 3%, D: 5%, E: 7%, vol/vol) on Y sperm sorting efficiency, based on the specific binding of Resiquimod (R848) to toll-like receptor (TLR)7/8 receptors on the tail of X sperm.

**Methods:**

The different concentrations of PVP were added to the R848 sperm sorting solution to facilitate the separation of Y sperm. Subsequently, the isolated sperm were subjected to quantification and motility assessment using computer-assisted semen analysis system. The X/Y sperm ratio is then analyzed by flow cytometry. The sorted sperm were evaluated for acrosomal and plasma membrane integrity. The spermatozoa were then subjected to immunofluorescent staining through immunofluorescence (IF) techniques, which preceded the quantification of the negative sperm rate. The proportion of male embryos was determined through embryonic sex identification after *in vitro* fertilization.

**Results:**

Flow cytometry analysis results showed that when the PVP concentrations were 3%, 5% and 7%, the proportion of Y sperm was not statistically significant, (p≥0.05). However, these percentages were significantly elevated compared to those obtained with 0% and 1% PVP concentrations (p<0.05). The IF staining results demonstrated that the proportion of TLR7/8-negative sperm remained statistically unchanged across PVP concentrations of 3%, 5%, and 7% (p≥0.05). However, these percentages were significantly elevated compared to those obtained with 0% and 1% PVP concentrations (p<0.05). The generation of male blastocysts was significantly higher at a PVP concentration of 3% compared to 0% and 1% (p<0.05), but showed no significant difference from 5% and 7% (p≥0.05).

**Conclusion:**

Selecting a 3% PVP concentration not only ensures sufficient sperm yield but also promotes effective selection of Y-sperm. These findings provide empirical evidence supporting the high-efficiency separation of X/Y sperm in livestock.

## INTRODUCTION

In contemporary animal husbandry practices, there is a discernible trend towards favoring the raising of animals of a specific gender. This preference is primarily driven by the specific requirements and goals of different types of farming. For example, in dairy farming, there is a preference for raising cows, while in beef cattle farming, there is a lean towards raising steers [1]. The fundamental approach to gender control in livestock is the separation of X/Y sperm. There are various techniques used for separating X/Y sperm in livestock, such as flow cytometry-based methods [2,3], X/Y sperm swim-up based methods [4], and pH gradient-based methods [5,6]. The most prevalent technique for sperm sorting in the current landscape of animal husbandry is flow cytometry [7], a method that has significantly improved the economic efficiency of animal husbandry. Despite its effectiveness, the main challenges with this technology are the high cost of flow cytometers and their limited availability [8]. In response to these challenges, we are conducting research to identify low-cost and highly effective alternatives to traditional flow cytometry, aiming to make gender control in livestock more accessible and economically viable.

In the quest to refine gender control in livestock, the exploration of novel methods for sorting X/Y sperm has seen significant advancements in recent years [9–11]. A notable breakthrough by Umehara et al [12] revealed that R848 can bind to toll-like receptor (TLR)7/8 on the tail of X sperm, which can effectively reduce the motility of X sperm, R848 as a TLR7/8 agonist, promotes the phosphorylation of nuclear factor (NF)-κB and glycogen synthase kinase (GSK)3α/β in X sperm, further inhibiting adenosine triphosphate (ATP) production via the GSK3α/β-hexokinase pathway, thereby suppressing the motility of X sperm. Ren et al [4] optimized the concentration of R848 in goat sperm medium, achieving a sorting rate of 80% for Y sperm through embryo sex determination, but the sorting efficiency for this method was found to be relatively low. The introduction of polyvinylpyrrolidone (PVP) aimed to leverage the binding capability of R848 to the TLR7/8 receptor on the tail of the X sperm, thus providing a novel approach to enhance the sorting efficiency of Y sperm [13].

PVP is a synthetic, water-soluble polymer that exhibits the common characteristics of water-soluble macromolecules. Its remarkable stability often renders it a preferred choice as a thickening agent [14,15]. PVP plays a crucial role in assisted reproductive technologies, particularly in intracytoplasmic sperm injection (ICSI) procedures [16,17], primarily for its immobilizing effect on sperm [18]. The PVP solution is still being used in assisted reproductive technology to this day [19]. By integrating R848 with a specific concentration of PVP can strategically target the motility of X sperm, which R848 has already compromised, to further limit their mobility. This methodological approach significantly enhances the selection process for Y sperm. In this study, the combination of R848 and PVP was employed to facilitate the efficient separation of Y sperm, and the quality of sperm after sexing was validated through computer-assisted semen analysis (CASA). The proportion of Y-chromosome-bearing sperm after sexing was further confirmed through flow cytometry, detection of sperm acrosome and plasma membrane integrity, immunofluorescent staining, *in vitro* fertilization, identification of embryonic gender. Thus, an optimal amount of PVP in the R848-treated sperm upstream selection medium was determined. This optimization can lead to the development of an upstream selection medium that is highly efficient in separating Y sperm and a convenient as well as efficient selection method could be achieved.

## MATERIALS AND METHODS

### Ethics statement

All animal experiments were conducted in accordance with the “Regulations and Guidelines for the Management of Experimental Animals” established by the Ministry of Science and Technology (Beijing, China, 2020 revision). This study was approved by the Institutional Animal Care and Use Committee of Tarim University, Xinjiang, China (protocol code DWBH20220101; approval date: 1 January 2022).

### Materials

#### Semen

Throughout the experiment, we used commercially available conventionally cryopreserved Simmental cattle semen from three bulls (ID: 41119897; 41119902; 41119915). A total of 75 frozen semen straws were consumed in this study. Commercially available frozen semen was 200 μL/tube frozen semen from straw, stored in liquid nitrogen. When thawing semen, we removed the frozen semen from liquid nitrogen and quickly placed it into a 38°C water bath. The samples were gently shaken for 30s; then, we tested the vitality, concentration, and X/Y sperm ratio. After thawing, the number of viable sperm should be ≥100 million/mL, with an X/Y sperm ratio 50:50 was verified using dual TaqMan qPCR. The probe for identifying X sperm is HPRT1: Probe: HEX-CCCACTGCATCAAGCCTGGTGTTAAA-TAMRA. The primers are: F: AGCAAGCAGCTGGGATATG, R: TGTCTC GGTGTATGGCTAGTA. The probe for identifying Y sperm is SRY: Probe: HEX-TAGAAATGTCAGTTGCTGCATT CCCGA-TAMRA. The primers are: F: GTGGCCAGCT GCTTAATAGA, R: AGGCTCGTAGTGCAAATGAA. The annealing temperature for both is 60°C. We collected the thawed semen into a centrifuge tube for later use.

#### Oocyte collectoon

Ovaries were provided by our local slaughterhouse and transported back to the laboratory within 1 h of collection (in an incubator set at 37°C). Then, we used a 10 mL syringe with a size 21-gauge needle to extract oocytes from follicles larger than 8 mm. In this experiment, a total of 2357 oocytes were collected.

All chemicals and reagents were purchased from Sigma-Aldrich China, unless otherwise specified. The preparation solutions used in this study were constituted according to the methods previously reported by Umehara et al [12], including TCM-199 oocyte aspiration fluid, bovine oocyte maturation culture medium, bovine embryo culture medium, basal media Stock A, Stock B, Stock C, and modified Human Tubal Fluid (mHTF) medium. The details regarding the selection solution used for Y sperm separation have been provided in the Supplement 1.

### Experimental design

**Figure 1:**
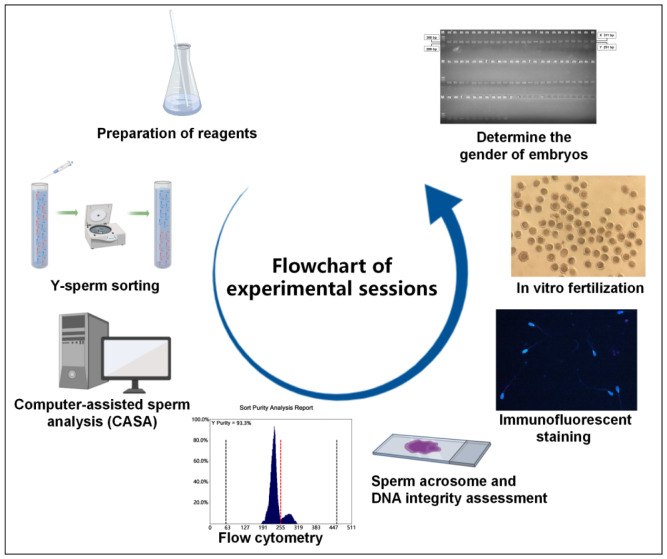
Flowchart of the experimental procedure.

In this study, varying concentrations of PVP (437190; Sigma-Aldrich China, Shanghai, China) were added to the upstream sorting solution of R848 (SML0196; Sigma-Aldrich China), with five gradient settings for the amount of PVP added, specifically A: 0%, B: 1%, C: 3%, D: 5%, and E: 7% (vol/vol). The sperm collected from the upper layer of each of the 6 groups was then quantitatively assessed after sexing. The concentration of motility characteristics of the sperm, including curvilinear velocity (VCL), straight-line velocity (VSL), and average path velocity (VAP), were analyzed using the CASA method. The X/Y sperm ratio is then analyzed by flow cytometry. Subsequently, the sorted spermatozoa were subjected to immunofluorescent staining, followed by the quantification of negative sperm rate. After *in vitro* fertilization, blastocyst formation rate was assessed, the identification of embryonic gender was utilized to determine the rate of male embryos to determine the proportion of Y sperm (as depicted in Figure 1, following the order of experiments conducted rather than the chronological order). The final determination of the optimal amount of PVP in the upstream sorting solution of R848. All experiments above were independently repeated at six times.

### Sperm sex selection using R848 and polyvinylpyrrolidone

**Figure 2:**
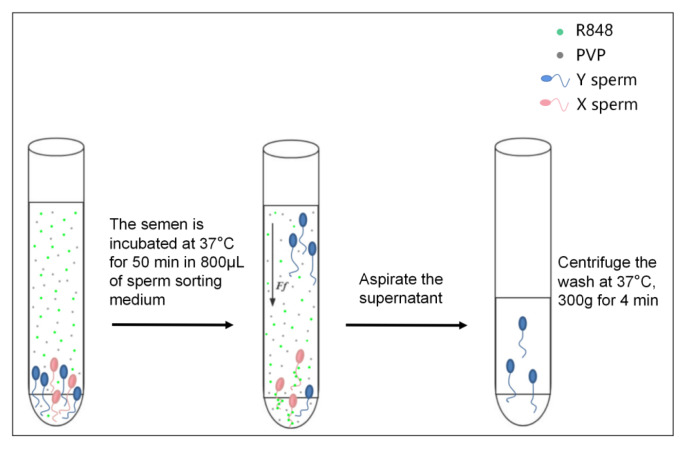
Semen upstream schematic.

To prepare for sorting, take a tube of cryopreserved regular semen and thaw it in 37°C water for 30 s. The thawed semen was then transferred into a 1.5 mL polyethylene (PE) tube. It was washed with 1 mL of mHTF culture medium, followed by two rounds of centrifugation (300 g/min, 5 min, 37°C) to remove the cryoprotectant. Thereafter, 0.2 mL of the washed semen was slowly injected into the bottom of a tube containing 0.8 mL of sperm sorting fluid (The sperm sorting fluid can be found in the Supplement 1), ensuring that the initial volume of semen was consistent across all experimental groups. These tubes were then incubated at 37°C in a 5% CO_2_ incubator for 50 min. Following the incubation, the upper layer 0.5 mL was carefully transferred into a new 1.5 mL PE tube. This sample was then subjected to centrifugation at 300×g for 4 min. After centrifugation, the resulting sperm pellet was gently resuspended in mHTF culture medium, preparing it for subsequent stages of the experiment. This procedure, including the resuspension of the sperm pellet is represented in Figure 2.

### Sperm motility performance assessment

Sperm motility parameters were analyzed by a computer-assisted sperm analysis (CASA) system (ML-500JZ; Mairong, Guangxi, China), which included an inverted microscope (TS100-F; Nikon, Tokyo, Japan). The semen samples treated with varying amounts of PVP sperm separation liquid, each 10μL (sperm count≈4.5×10^5^), were assessed, and slowly injected the sperm into a sample chamber with a depth of 20 μm without generating bubbles. The slide was placed on a microscope stage at 37°C. Then adjusted the brightness of the microscope illumination and display it on the display screen. Sperms can be clearly seen, and the sperm was captured at a frequency of 60 Hz (0.5 seconds, 30 frames). Measurements of sperm concentration, the proportion of motile sperm, and five CASA parameters were used for further analysis. The CASA parameters included three measures of sperm velocity (VCL, VSL and VAP). Sperm were observed from at least five randomly selected fields.

### Detection of sperm acrosome and plasma membrane integrity

The integrity of sperm acrosome was evaluated using fluorescein isothiocyanate (FITC)-peptide nucleic acid (PNA)/4′,6-diamidino-2-phenylindole (DAPI) staining method (MP6327; MX4208; Maokang Biotechnology Co., Ltd., Shanghai, China). Firstly, 20 μL of selected sperm from different groups were placed on slides, air-dried, then fixed in 2% paraformaldehyde solution (MM1515; Maokang Biotechnology Co., Ltd.) for 10 min followed by air-drying. 2 mL of phosphate-buffered saline (PBS) was added on the slides, incubated for 10 min, repeated twice to wash away the paraformaldehyde fixative. Subsequently, 30 μL of FITC-PNA working solution was applied to stain the sperm sample, ensuring even coverage, followed by incubation in the dark at 37°C for 30 min, washing with PBS three times to remove excess stain, then adding 30 μL of DAPI staining solution uniformly on the sample slides, ensuring coverage, incubating in the dark at room temperature for 5 to 10 min, covering with coverslip, then quickly observing and capturing images under a fluorescence microscope (Nikon Ti2-U, Nikon), ensuring avoidance of light exposure throughout. Each field of view should not contain fewer than 200 sperm for counting. Sperm with intact and bright green fluorescence in the anterior part of the acrosome were considered as intact acrosome sperm, while those with incomplete or absent fluorescence in the anterior part were considered as acrosome damaged sperm. Complete darkness was maintained during photography. The acrosome integrity rate was calculated using the formula: Acrosome integrity rate = Number of acrosome intact sperm / Total number of sperm×100%. The analysis of sperm membrane integrity was performed using SYBR 14/propidium iodide (PI) staining method (MX4239; MX4205; Maokang Biotechnology Co., Ltd.), which followed the same protocol as FITC-PNA/DAPI staining. Sperm appearing green indicated live sperm, while red indicated loss of membrane integrity. The membrane integrity rate was calculated using the formula: Membrane integrity rate = Number of membrane intact sperm / Total number of sperm×100%.

### Immunofluorescence staining

The sorted bovine spermatozoa were spread on glass slides and air-dried, fixed in 100% methanol for 10 min, washed thrice with PBS for 5 min each, followed by permeabilization with 0.5% Triton X-100 at room temperature for 30 min, and washed three times with PBS for 5 min each. Glass slides were incubated with diluted rabbit polyclonal antibody TLR7 (1:1500) (DF6173; Affinity Biosciences Co., Ltd., Changzhou, China) and rabbit polyclonal antibody TLR8 (1:1500) (DF6426, Affinity Biosciences Co., Ltd.), followed by overnight incubation at 4°C. After incubation, the samples were washed with PBS. Sperm incubated with TLR7 were further incubated with FITC-conjugated goat anti-rabbit IgG (1:1000; ab6717; Abcam, Shanghai, China) for 2 hours, and sperm incubated with TLR8 were also incubated with Cy3-conjugated goat anti-rabbit IgG (1:1000; ab6939; Abcam) for 2 hours. After washing with PBS, the sperm were stained with 1 μg/mL DAPI (MX4208; Maokang Biotechnology Co., Ltd.) and observed and imaged using a fluorescence digital microscope (Nikon Ti2-U, Nikon). The staining of the sperm was visualized with FITC excited at 528 nm and Cy3 excited at 565 nm. Sperm were examined from at least five randomly selected fields of view.

### *In vitro* fertilization

Within 1 h post-slaughter, the ovaries of cattle were stored in a physiological saline solution at 37.0°C and transported from the abattoir to the laboratory. The follicles (with a diameter of 2–8 mm) were manually aspirated using a 10 mL syringe filled with 2 mL of TCM-199 oocyte aspiration fluid. Oocytes surrounded by at least three layers of compact cumulus granulosa cells (COCs) were selected for *in vitro* maturation (IVM). These oocytes were washed thrice in TCM-199 medium supplemented with HEPES buffer, followed by two additional washes in TCM-199 containing NaHCO_3_. The COCs were then transferred to a culture dish containing 0.8 mL of bovine oocyte maturation medium and cultured in a 38.5°C and 5% CO_2_ incubator for 24 h. After 24h maturation period, cumulus cells were dissociated by pipetting the COCs in 0.1% (vol/vol) hyaluronidase at 38.5°C for 1 min. The mature oocytes, identified by the presence of the first polar body and absence of cumulus cells, were randomly allocated to the experimental groups. The sorted sperm were then gently added to the culture dish containing COCs in the form of a 30 μL droplet (sperm count≈1.35×10^6^). This setup was incubated for 24 h in a culture chamber set at 38.5°C and 5% CO_2_. Following fertilization, the embryos were transferred to a dish containing embryo culture medium and cultured in a 38.5°C, 5% CO_2_ incubator until they reached the blastocyst stage.

### Identification of embryonic gender

The blastocysts were washed in PBS before being individually placed into 0.2 ml nuclease-free centrifuge tubes. To each tube, 5 μL of Genome extraction solution (50 mmol/L Tris-HCl, pH = 8.0, 0.5% Triton X-100, 1 mg/mL Proteinase K) was added. Each test tube contained one blastocyst, followed by lysis. The 5 μL of Genome extraction solution obtained post-lysis was used as the template for polymerase chain reaction (PCR). The primers targeting the amelogenin (*AMELY*) gene, detailed in Table 1, were designed to perform PCR-based gender determination on the individual embryos. For the second round of PCR, 1 μL of the product from the first round was used as the template. The nested PCR procedures for both the first and second rounds were identical, ensuring consistency in the amplification process. Subsequently, the PCR products were subjected to 2% agarose gel electrophoresis at 100 V for 20 min, followed by the visualization and photography using a gel imaging system.

### Statistical analysis

Statistical graphs were generated using GraphPad Prism software version 8.0 (GraphPad Software, Inc., San Diego, CA, USA). The data was presented as mean ± standard deviation, based on a minimum of three independent experiments. The comparison of variables between different groups was conducted using Student’s t-test (two-tailed). When multiple comparisons were necessary, analysis of variance (ANOVA) was employed, Pairwise comparison was done using Tukey honest significant difference test. A p-value of less than 0.05 was deemed to indicate statistical significance.

## RESULTS

### The analysis findings from the computer-aided analysis system

**Figure 3:**
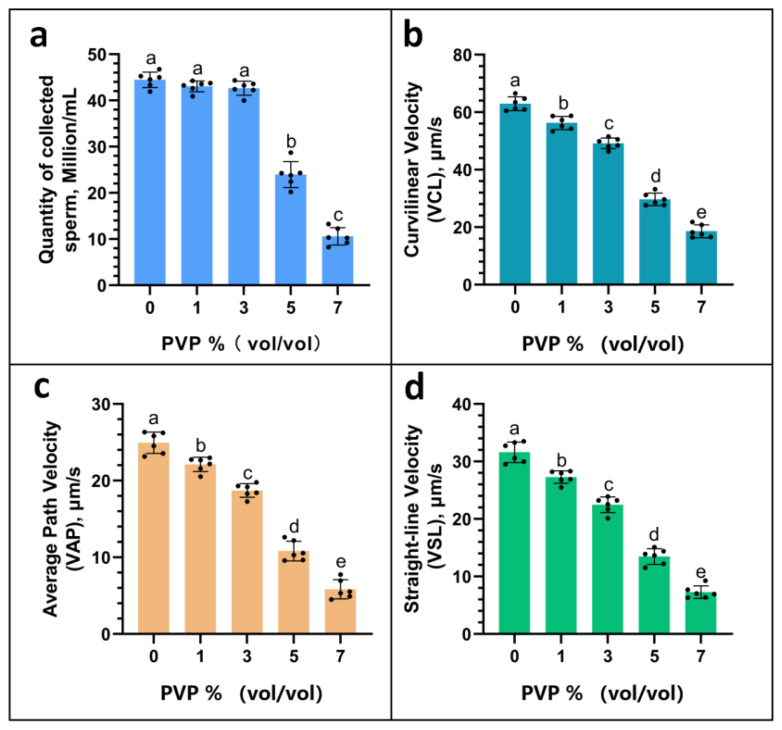
The impact of various sperm parameters after addition of different concentrations of PVP. (a) The quantity of the collected sperm. (b) Curvilinear velocity. (c) Average path velocity. (d) Straight-line velocity. In the statistical chart, the same letter indicates that there is no significant difference p≥0.05, and different letters indicate a significant difference p<0.05. PVP, polyvinylpyrrolidone.

In the results obtained from computer-aided sperm analysis (Figure 3), the quantities of sperm collected in the upper layer when PVP concentrations were A: 0%, B: 1%, and C: 3% did not significantly differ, with values reported as (A: 44.49±1.66, B: 42.72±1.71, C: 42.59±1.61 million/mL) (p≥0.05). However, these values are significantly higher than when the amount of PVP added is D: 5% and E: 7%, resulting in the collection of sperm in the upper layer (D: 23.97±2.24, E: 10.60±1.80, Million/mL) (p<0.05). Moreover, during the analysis of sperm motility parameters (as shown in Figures 3B–3D), an increase in the PVP concentration was associated with a decline in VCL, VSL, and VAP values, indicating a decreasing trend in sperm motility. Furthermore, the differences in VCL, VSL, and VAP across the groups were statistically significant (p<0.05), demonstrating the impact of PVP concentration on sperm motility characteristics.

### Flow cytometry analysis results

**Figure 4:**
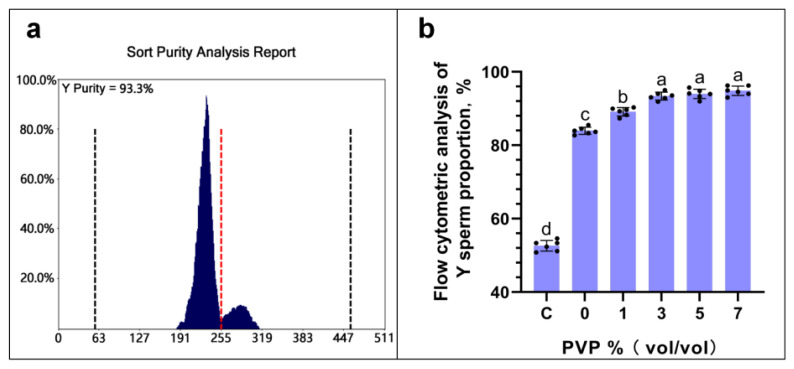
Flow cytometry analysis results. Figure 4a shows the results of flow cytometric analysis with a PVP concentration of 3%, while Figure 4b displays the results of flow cytometric analysis at varying PVP concentrations. In the statistical chart, the same letter indicates that there is no significant difference, p≥0.05, and different letters indicate a significant difference, p<0.05. PVP, polyvinylpyrrolidone.

The flow cytometry analysis results are shown in Figure 4. Figure 4A which pertains to the sperm flow cytometry analysis with the addition of 3% PVP in the sorting solution. Results from other experimental groups can be found in the Supplement 2. The flow cytometry analysis indicates that, as shown in Figure 4b, there were no significant differences in the proportions of Y sperm at PVP addition levels C: 3%, D: 5%, and E: 7% (p≥0.05), with values of (C: 93.07±1.26%, D: 94.26±1.35%, E: 95.83±1.09%), all of which were significantly higher than the PVP addition levels A: 0% and B: 1% (A: 83.65±1.16%, B: 88.52±1.33%) (p<0.05).

### Assessment of sperm acrosomal and membrane integrity

**Figure 5:**
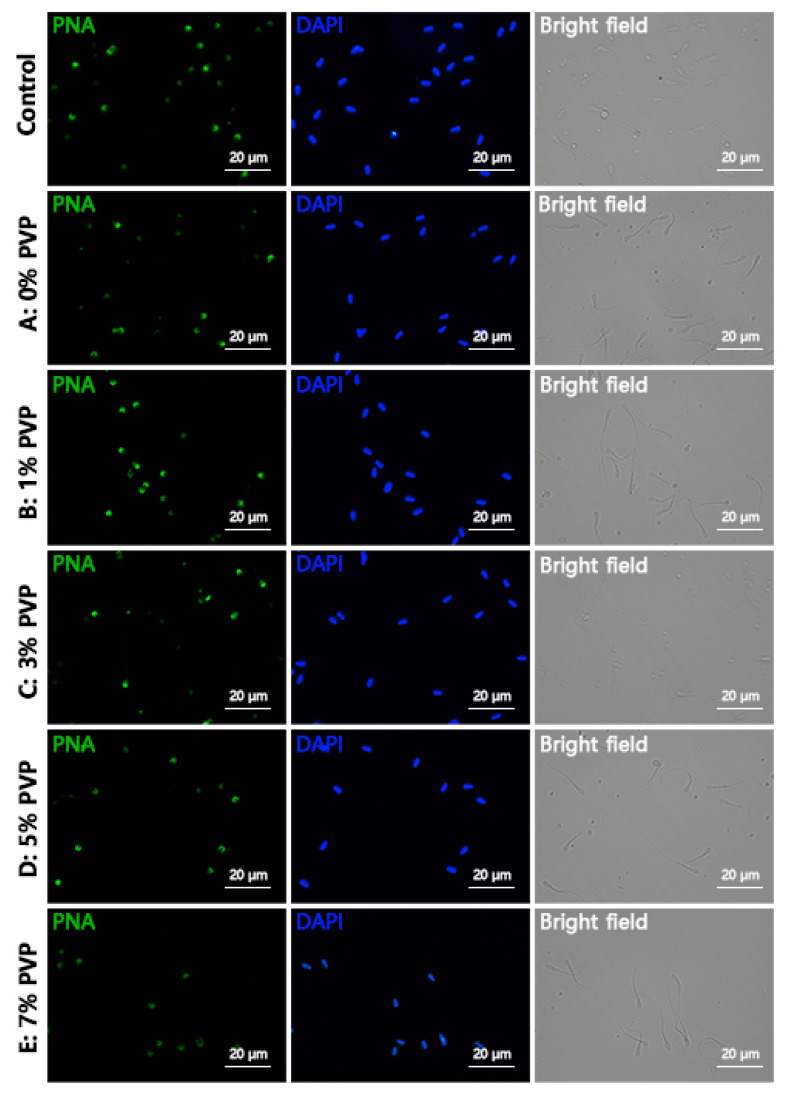
Assessment results of sperm acrosomal integrity. Control indicates sperm that were not treated with R848 and PVP. Groups A–E were all treated with RB48 and subjected to different concentrations of PVP treatment. Green staining represents FITC-PNA, indicating intact acrosomes, while blue staining represents DAPI, which stains all nuclei. The scale bar in all images is 20 μm. PVP, polyvinylpyrrolidone; FITC, fluorescein isothiocyanate; PNA, peptide nucleic acid; DAPI, 4′,6-diamidino-2-phenylindole.

**Figure 6:**
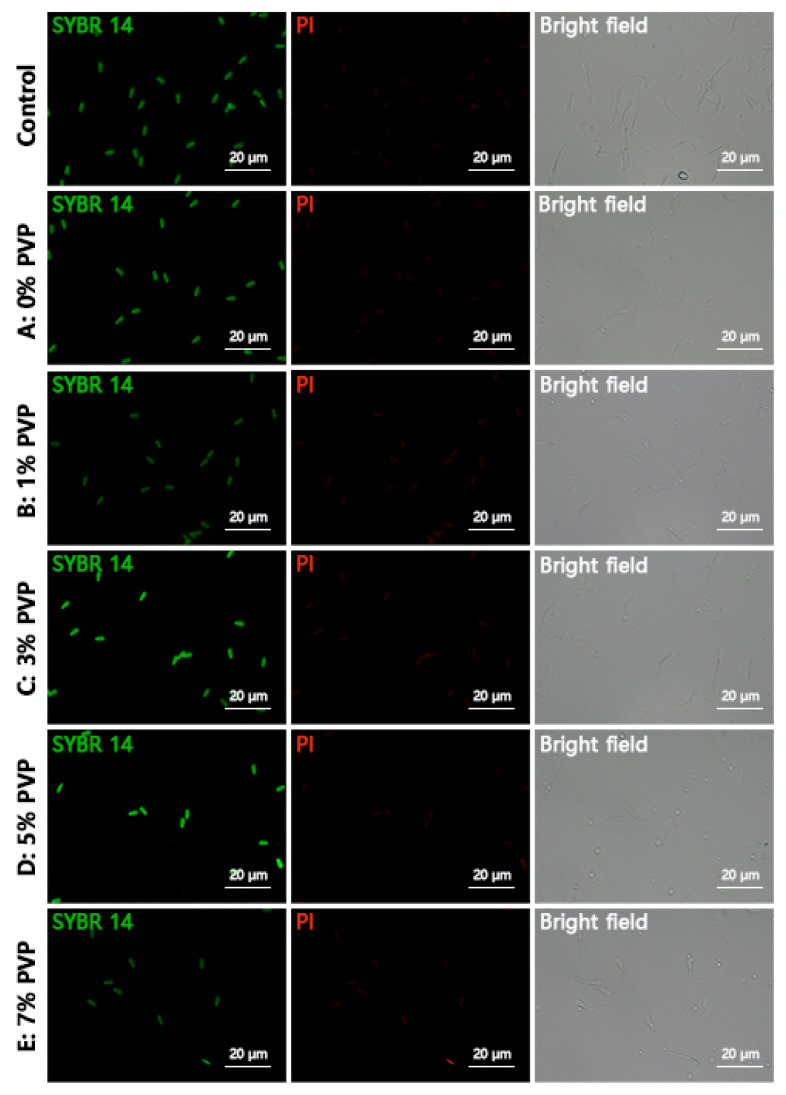
Assessment results of sperm membrane integrity. Control indicates sperm that were not treated with R848 and PVP. Groups A–E were all treated with RB48 and subjected to different concentrations of PVP treatment. Green staining represents SYBR 14, indicating live sperm, while red staining represents PI (propidium iodide), indicating sperm membrane damage, which stains all nuclei. The scale bar in all images is 20 μm. PVP, polyvinylpyrrolidone.

**Figure 7:**
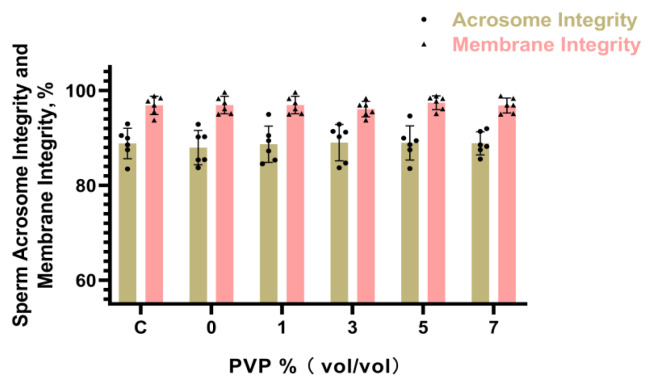
Statistical results of sperm acrosomal and membrane integrity. Absence of letters indicates no significant differences between treatments.

The integrity of sperm acrosome as shown in Figure 5, with the Control group representing sperm not subjected to R848 and PVP treatment. Groups A, B, C, D, E were treated with R848 and then with 0%, 1%, 3%, 5%, 7% PVP, respectively. Sperm with intact acrosome were stained green with FITC-PNA, while acrosome-deficient sperm remained uncolored. The integrity of sperm plasma membrane is shown in Figure 6, following the same treatment grouping as the sperm acrosome integrity assessment. Live sperm were stained green with SYBR 14, while sperm with damaged plasma membrane were stained red with PI. Statistical analysis of the results revealed that the impact of R848 and PVP treatment on sperm acrosome and plasma membrane integrity was not significantly different (p≥0.05) (Figure 7). The results for acrosome integrity were as follows: Control: 88.84±3.11%, A: 87.99± 3.41%, B: 88.25±2.81%, C: 89.36±2.95%, D: 87.52±3.02%, E: 87.95±3.31%. The results for plasma membrane integrity were as follows: Control: 96.82±1.93%, A: 96.35±1.84%, B: 95.93± 1.81%, C: 96.25±1.63%, D: 97.23±1.47%, E: 96.82±1.58%.

### Localisation of TLR7/8 in sperm

**Figure 8:**
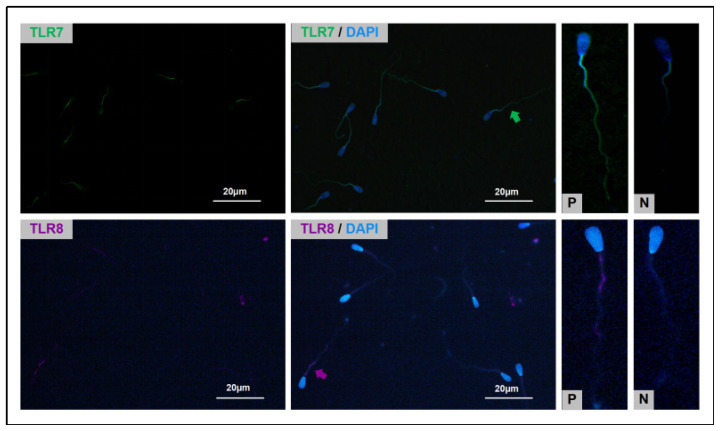
Immunofluorescence staining results. The green arrow in the figure indicates TLR7 receptors fluorescence staining; the rose red arrow indicates TLR8 receptors fluorescence staining. TLR, toll-like receptor.

**Figure 9:**
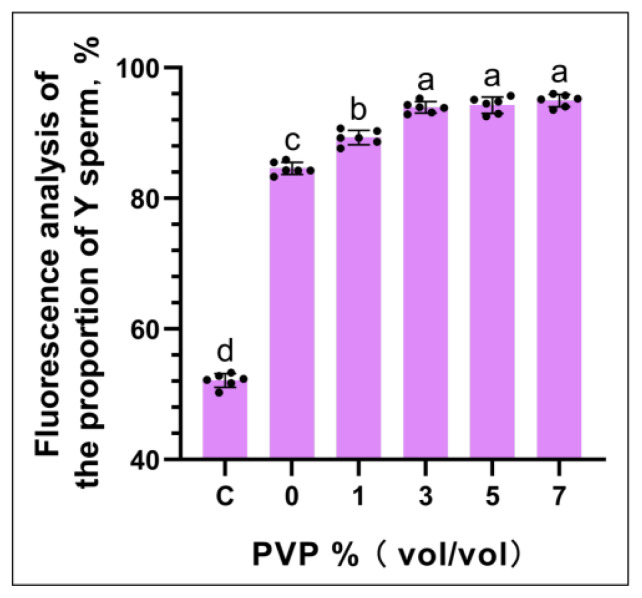
Immunofluorescence statistical results. In the statistical chart, the same letter indicates that there is no significant difference, p≥0.05, and different letters indicate a significant difference, p<0.05.

As shown in Figure 8, TLR7 is primarily distributed along the entire tail of the sperm, while TLR8 is mainly distributed in the anterior and midpiece of the sperm tail. After conducting immunofluorescent staining, the distribution and ratio of TLR7/8-negative sperm within the entire sperm population were analyzed, as depicted in Figure 9. Immunofluorescence staining analysis results showed that when the PVP concentrations were C: 3%, D: 5%, and E: 7% (C: 93.83±0.86%, D: 94.61±1.41%, E: 96.18±1.22%), the percentage difference of tlr7/8 negative sperm was not significant (p≥0.05). However, these values were significantly higher than those at PVP concentrations A: 0% and B: 1% (A: 84.30±1.32%, B: 89.22± 1.17%) (p<0.05), indicating that the increase in PVP concentration enhances the selection efficiency for tlr7/8 negative (including Y) sperm.

### *In vitro* fertilization and embryo gender identification analysis

**Figure 10:**
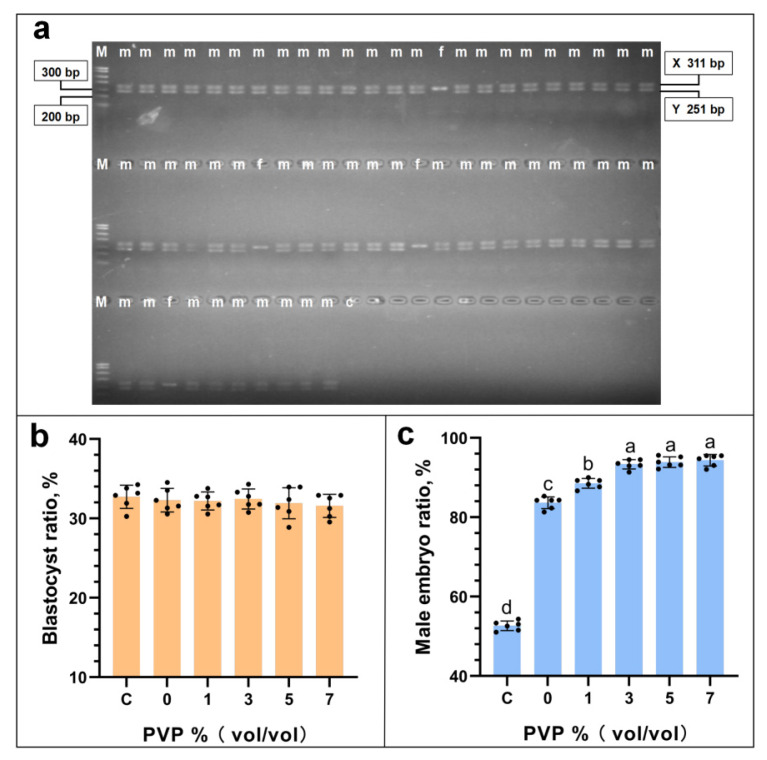
Identification of embryonic gender results. Figure 7a represents the gel electrophoresis image of embryonic cell identification at a PVP concentration of 3%. The “M” in the image represents the DNA marker (700 bp), whereas “c” represents the negative control. The two bands in the image represent the PCR results of the male embryos (represented by the letter “m”), but the single band represents the PCR result of a female embryo (represented by the letter “f”). Figure 7b represents the statistical results of blastocyst formation from *in vitro* fertilization. Figure 7c represents the statistical results of embryonic cell identification for different experimental groups. In the statistical chart, the same letter indicates that there is no significant difference, p≥0.05, and different letters indicate a significant difference, p<0.05. PVP, polyvinylpyrrolidone; PCR, polymerase chain reaction.

The gel electrophoresis gel image for embryo gender identification has been shown in Figure 10A. This specific gel image pertains to embryos derived from *in vitro* fertilization using sperm selected with the addition of 3% PVP in the sorting solution. The gel electrophoresis images for embryo gender identification from other experimental groups are provided in the Supplement 2. The results of in vitro fertilization and embryo gender identification (Figures 10A,10C) showed that the rate of blastocyst formation resulting from *in vitro* fertilization exhibited no significant variation with increasing amounts of PVP (p≥0.05), indicating that the concentration of PVP used in sperm sorting does not adversely affect the subsequent ability of the fertilized embryos to develop into blastocysts. The results of embryonic sex determination show that at PVP concentrations C: 3%, D: 5%, and E: 7%, the production rates of male embryos did not differ significantly, being (C: 93.35±1.10%, D: 94.21±1.03%, E: 95.62±1.35%) (p≥0.05). However, these rates were significantly higher than those at PVP concentrations A: 0% and B: 1% (A: 83.97±1.51%, B: 88.42±0.78%) (p<0.05).

## DISCUSSION

This study investigates the precise interaction of R848 with TLR7/8 receptors found on sperm tails to determine the ideal concentration of PVP in the sperm sorting solution for effectively separating Y sperm. By employing CASA, flow cytometry, immunofluorescent labeling, statistical evaluation of embryo sex determination through *in vitro* fertilization, it has been determined that the addition of 3% PVP to the R848 sperm selection solution ensures the quantity of sorted sperm while effectively increasing the sorting rate of Y sperm.

The binding of R848 to the TLR7/8 receptors on the tail of the X sperm restricts the motility of the X sperm, primarily because of the observed increase in phosphorylated GSK3 α/β and NF-κB following the activation of TLR7/8 in X sperm. This results in diminished mitochondrial function, reduced ATP levels, and lower sperm vitality [20]. In this study, when the PVP concentration was 0%, our findings were consistent with those reported by Umehara et al [21], the distinction being that we have exclusively validated the proportion of Y sperm in the upper layer. To enhance the efficiency of Y sperm separation, PVP was added into the upper fluid with R848 in this experiment.

The use of PVP solution in ICSI dates back to 1995, where it was employed as a means to immobilize sperm, primarily aiming to slow down sperm motility [22,23]. The reason why PVP can influence the separation of X/Y sperm is that in this experiment, after the TLR7/8 of X sperm were activated by R848, the motility of X sperm was somewhat diminished. As the concentration of PVP increased, the fluid became progressively more viscous, making it even more difficult for the already low-motility X sperm to swim. This ultimately led to the separation of X/Y sperm. We found that as the concentration of PVP increased, the volume of sperm collected decreased. However, when comparing the quantities of sperm collected at different PVP concentrations—A: 0%, B: 1%, and C: 3%—the differences were not statistically significant (p≥0.05). However, these values were significantly higher than when the amount of PVP added is D: 5% and E: 7%, resulting in the collection of sperm in the upper layer (p<0.05). As the concentration of PVP increased, there was a noticeable decline in the motility parameters of the collected sperm, including VCL, VSL, and VAP. These values (VCL, VSL, VAP) across each group differed significantly (p<0.05), suggesting that higher amounts of PVP might have restricted sperm motility to some extent. Conversely, with higher PVP concentrations, the rate of male embryos produced through *in vitro* fertilization exhibited an upward trend. Interestingly, at PVP concentrations of C: 3%, D: 5%, and E: 7%, the variation in the percentage of male embryos was not statistically significant (p≥0.05), although these rates were significantly higher compared to when the PVP concentration was at A: 0% and B: 1% (p<0.05). Additionally, the elevated success rate of male embryos observed surpasses the findings of the Umehara et al study [21], indicating that PVP indeed plays a role in limiting the motility of the less vigorous X sperm. This is further supported by the success rate of male calves born through artificial insemination, which aligns with the notion that PVP’s effect on sperm motility selectively favors the Y sperm. An examination of the relationship between the volume of sperm collected and the success rate of producing male embryos via *in vitro* fertilization revealed that a 3% concentration of PVP strikes an optimal balance. This concentration maintains sperm quantity while significantly enhancing the purity of separated Y sperm, marking it as the most advantageous for use. While some researchers argue that both the quantity of PVP added and its exposure duration may have adverse effects on sperm [24,25], yet the sperm obtained in this experiment showed no adverse effects from PVP exposure, likely due to the relatively low concentration utilized.

## CONCLUSION

The findings indicated that as the concentration of PVP in the R848 upper solution was increased, the quantity of sperm collected decreased, accompanied by a declining trend in motility parameters such as VCL, VSL, and VAP. Concurrently, there was an observed increase in the production of male embryos. In conclusion, the addition of 3% PVP to the upstream R848 solution can facilitate the collection of a greater quantity of sperm and effectively enhance the efficiency of Y sperm separation.

## Figures and Tables

**Table 1:** The primers used for nested PCR

Primer	Sequence form 5′ to 3′	Annealing temperature °C	Fragment length	Reference sequence
*AMELY -1*	F: CATGGTGCCAGCTCAGCAG	62	X: 349 bp	NM_174240.2
	R: CCGCTTGGTCTTGTCTGTTGC		Y: 289 bp	
*AMELY -2*	F: CAGCAACCAATGATGCCAGTTC	62	X: 311 bp	
	R: GTCTTGTCTGTTGCTGGCCA		Y: 251 bp	

PCR, polymerase chain reaction.

## SUPPLEMENTARY MATERIAL

**Supplement 1**. Instructions for preparing the experimental solutions used in the study. **Supplement 2**. Flow cytometric analysis results of different experimental groups.
