# Supplementary material for: Polyvinylpyrrolidone can effectively improve the efficiency of resiquimod in sorting bovine Y sperm
Source: Anim Biosci. 2025 Mar 31;38(9):1881–93. doi: 10.5713/ab.24.0738 (PMC12415464; doi:10.5713/ab.24.0738)
Supplement: Supplementary file 1 [file ab-24-0738-Supplementary.pdf]

Supplementary Material

Supplement 1. Instructions for preparing the experimental solutions used in the study.

Stock A

Dissolve 2.969 g of NaCl, 0.1748 g of KCl, 0.3778 g of $CaCl_2 \cdot 2H_2O$, 0.0272 g of KH_2PO_4, 0.0246 g of $MgSO_4 \cdot 7H_2O$ and 0.0002 g phenol red in 40 mL of ddH_2O. Upon dissolution, adjust the volume to 50 mL with ddH_2O. Store at 4 °C and use within 1 week.

Stock B

Dissolve 1.0500 g of $NaHCO_3$ in 40 mL of ddH_2O. Upon dissolution, adjust the volume to 50 mL with ddH_2O. Store the solution at 4 °C and use within 1 week.

Stock C

Dissolve 0.0250 g of D-glucose, 0.0018 g of sodium pyruvate, 0.0025 g of streptomycin, 0.0031 g of penicillin G and 174 μL of sodium DL-lactate in 30 mL of ddH_2O. Store the solution at 4 °C and use within 1 week.

mHTF medium

5 mL Stock A, 5 mL Stock B, 30 mL Stock C, 250 mg BSA, 40 μL 500 mM creatine solution were mixed. Filter through a 0.45 μm membrane. Store at 4 °C and use within 1 week.

R848 sperm selection solution

Mix 40 mL of mHTF medium with 0.4 μM R848. Filter through a 0.45 μm membrane. Store at 4 °C and use within 1 week.

Sperm sorting solution with different concentrations of PVP

A: 0%：mHTF medium＋0 % (vol / vol) PVP.

B: 1%：mHTF medium＋1 % (vol / vol) PVP.

C: 3%：mHTF medium＋3 % (vol / vol) PVP.

D: 5%：mHTF medium＋5 % (vol / vol) PVP.

E: 7%：mHTF medium＋7 % (vol / vol) PVP.

Prepare the culture medium the day before use and equilibrate in a 37 °C CO_2 incubator for at least 2 h.

Extract oocyte fluid

Add 0.0350 g of heparin sodium and 0.050 g of BSA to 50mL of TCM-199, mix thoroughly, and filter the culture medium with a 0.45 μm filter. Store at 4 °C and use within 2 weeks.

Maturation medium for bovine oocytes

Add 0.02 IU/mL FSH, 5% (vol/vol) FBS, 1 μg/mL E_2, and 0.2 mM sodium pyruvate to TCM-199. Store at 4 °C and use within 1 week.

Culture medium for bovine embryos

Add 2% (vol/vol) essential amino acids (BME), 1% (vol/vol) MEM, and 5% (vol/vol) fetal bovine serum (FBS) to TCM-199. Store at 4 °C and use within 1 week.

Supplement 2. Flow cytometric analysis results of different experimental groups.

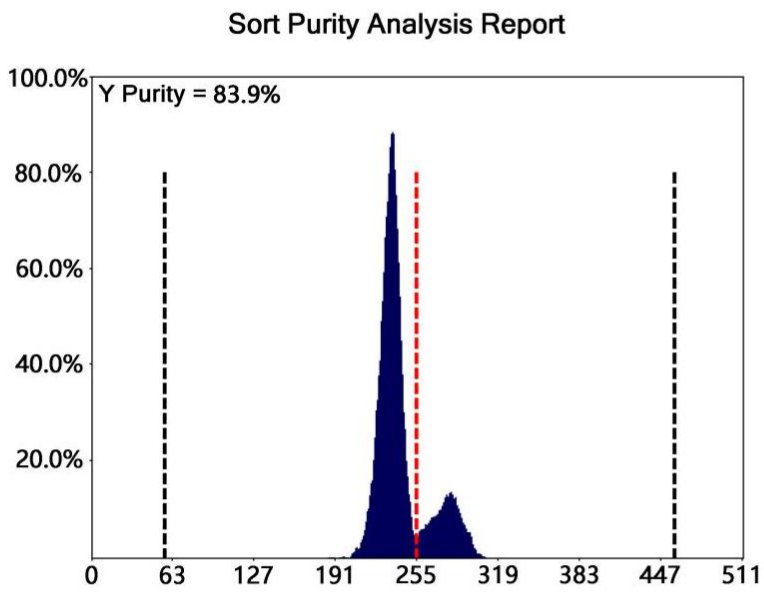

Fig.1 Results of flow cytometric analysis （PVP=0%）

Note: The concentration of PVP added is 0%. Flow cytometric analysis results show that the Y sperm ratio is 83.9%.

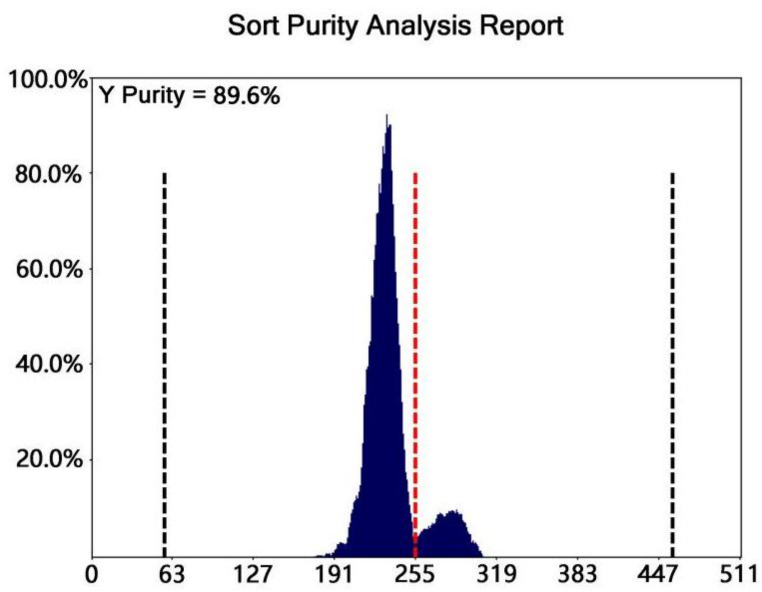

Fig.2 Results of flow cytometric analysis （PVP=1%）

Note: The concentration of PVP added is 1%. Flow cytometric analysis results show that the Y sperm ratio is 89.6%.

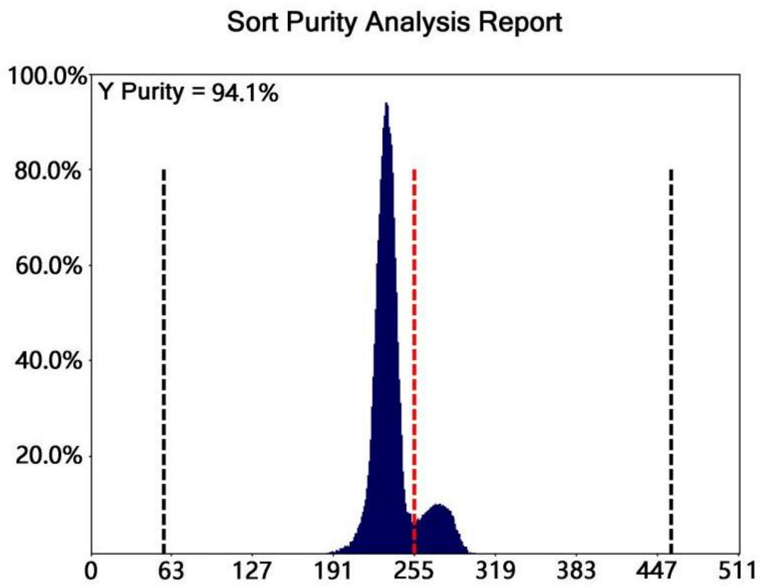

Fig.3 Results of flow cytometric analysis （PVP=5%）

Note: The concentration of PVP added is 5%. Flow cytometric analysis results show that the Y sperm ratio is 94.1%.

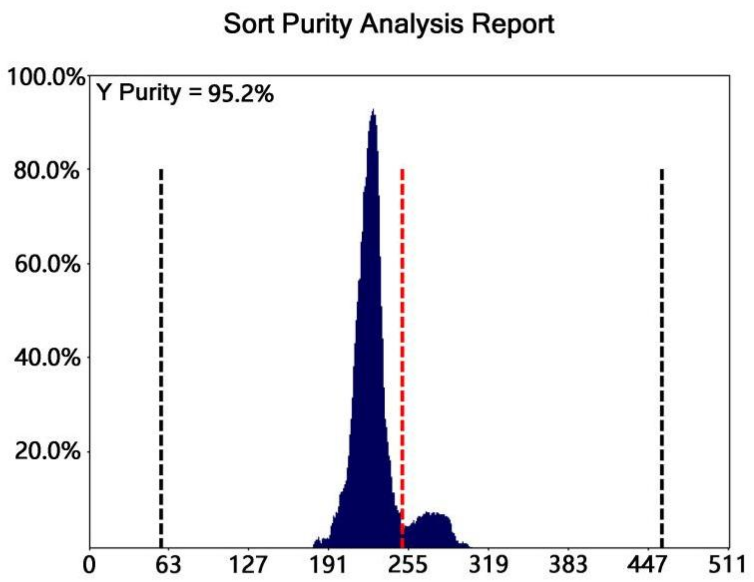

Fig.4 Results of flow cytometric analysis （PVP=7%）

Note: The concentration of PVP added is 7%. Flow cytometric analysis results show that the Y sperm ratio is 95.2%.

Electrophoresis gel images from different experimental groups.

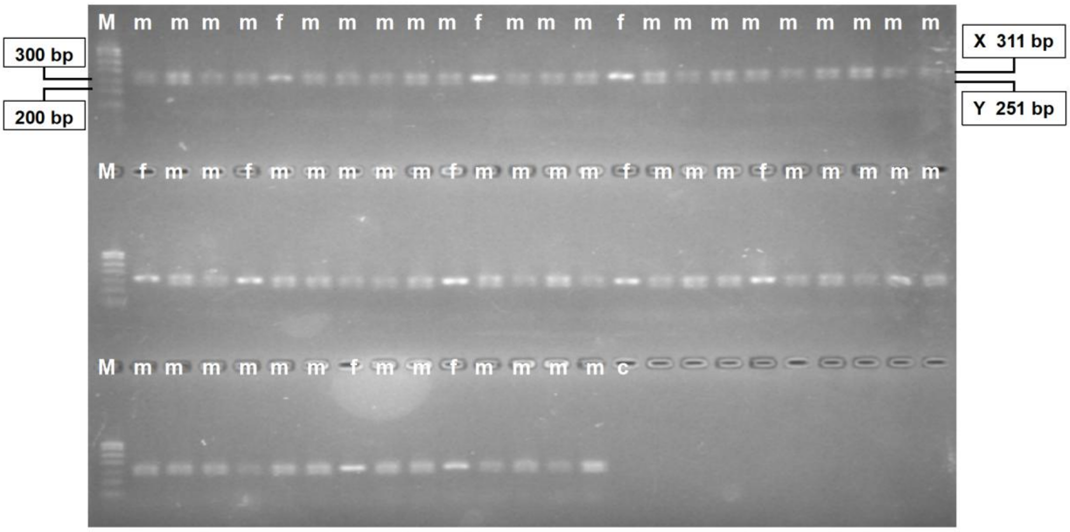

Fig.5 Results of embryo gender identification （PVP=0%）

Note: The addition of PVP at a concentration of 0% resulted in a male embryo rate of 83.87%.

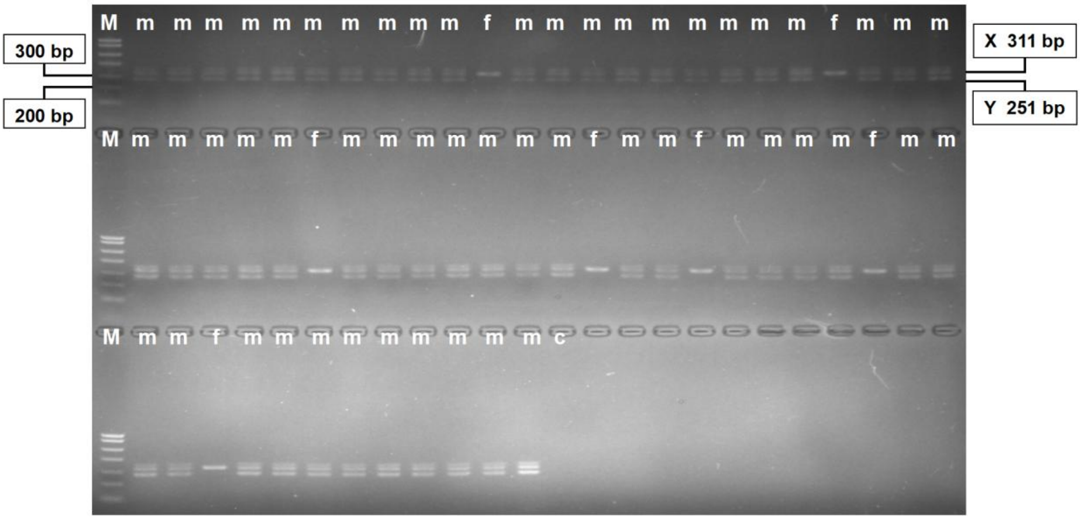

Fig.6 Results of embryo gender identification （PVP=1%）

Note: The addition of PVP at a concentration of 1% resulted in a male embryo rate of 88.33%.

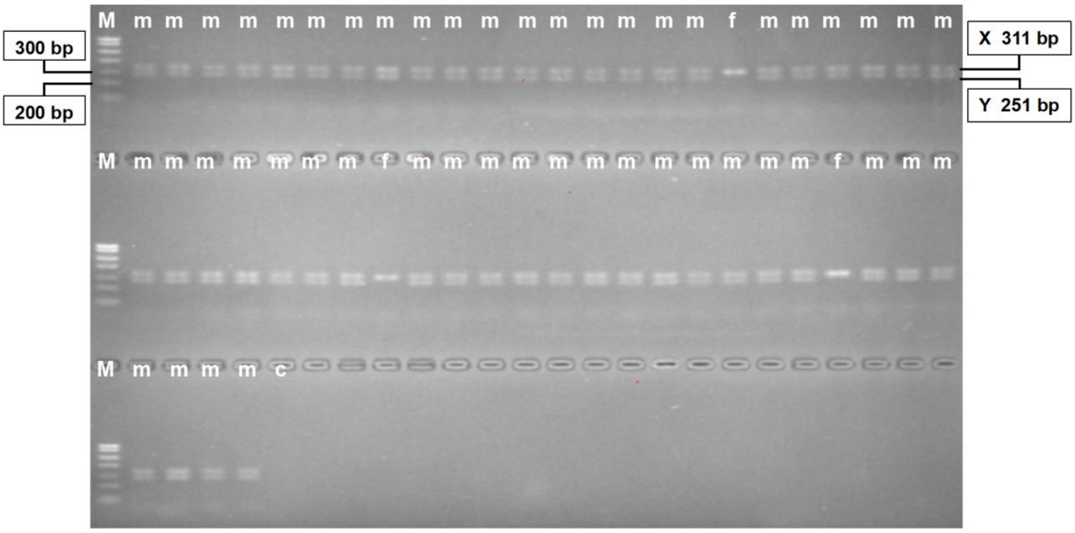

Fig.7　Results of embryo gender identification　（PVP=5%）

Note: The addition of PVP at a concentration of 5% resulted in a male embryo rate of 94.23%.

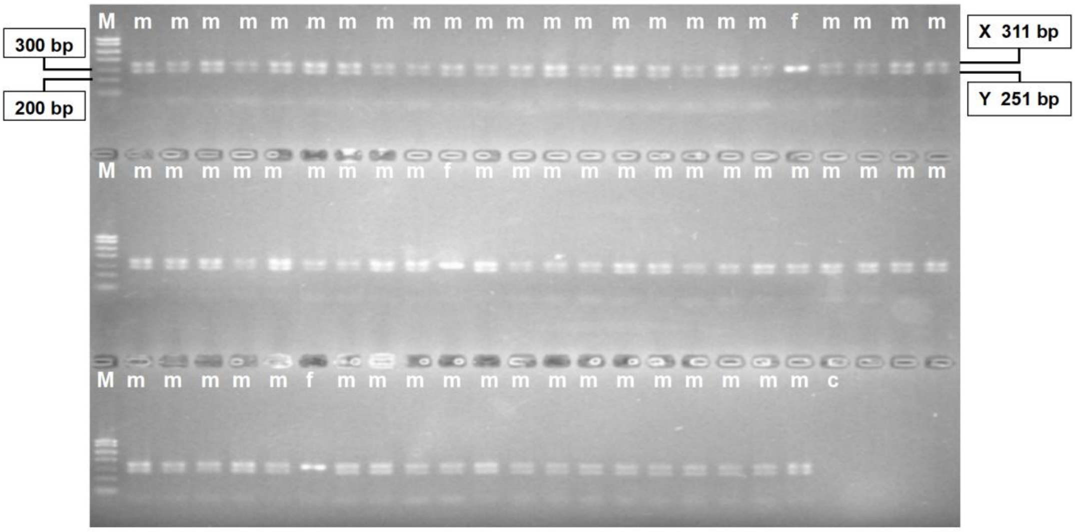

Fig.8　Results of embryo gender identification　（PVP=7%）

Note: The addition of PVP at a concentration of 7% resulted in a male embryo rate of 95.58%.
